# Supplementary material for: Characterization of N-glycosylation and its functional role in SIDT1-Mediated RNA uptake
Source: J Biol Chem. 2024 Jan 16;300(2):105654. doi: 10.1016/j.jbc.2024.105654 (PMC10850970; doi:10.1016/j.jbc.2024.105654)
Supplement: Supplemental Figs. S1−S6 [file mmc3.pdf]

Characterization of *N*-glycosylation and its Functional Role in SIDT1-Mediated RNA Uptake

Tingting Yang[1]*, Haonan Xiao[1]*, Xiulan Chen[2,3]*, Le Zheng[1], Hangtian Guo[1], Jiaqi Wang[1], Xiaohong Jiang[1], Chen-Yu Zhang[1,4]†, Fuquan Yang[2,3]†, Xiaoyun Ji[1,4,5,6]†

[1]The State Key Laboratory of Pharmaceutical Biotechnology, School of Life Sciences, Nanjing University, Nanjing, Jiangsu 210023, People's Republic of China.
[2]Key Laboratory of Protein and Peptide Pharmaceuticals & Laboratory of Proteomics, Institute of Biophysics, Chinese Academy of Sciences, Beijing 100101, China.
[3]University of Chinese Academy of Sciences, Beijing 100149, China.
[4]Chemistry and Biomedicine Innovation Center (ChemBIC), Nanjing University, Nanjing, Jiangsu 210023, People's Republic of China.
[5]Institute of Artificial Intelligence Biomedicine, Nanjing University, Nanjing, Jiangsu 210023, People's Republic of China.
[6]Engineering Research Center of Protein and Peptide Medicine, Ministry of Education, China.

* These authors contributed equally to this work.

To whom correspondence should be addressed. E-mail: xiaoyun.ji@nju.edu.cn (X.J.), fqyang@ibp.ac.cn (F.Y.), cyzhang@nju.edu.cn (C.Z.)

SUPPORTING INFORMATION

Supplementary figures S1-S6

Supplementary Table S1 (separate excel file): Analysis related to Figure 1D.

Supplementary Table S2 (separate excel file): Analysis related to Figure 1E.

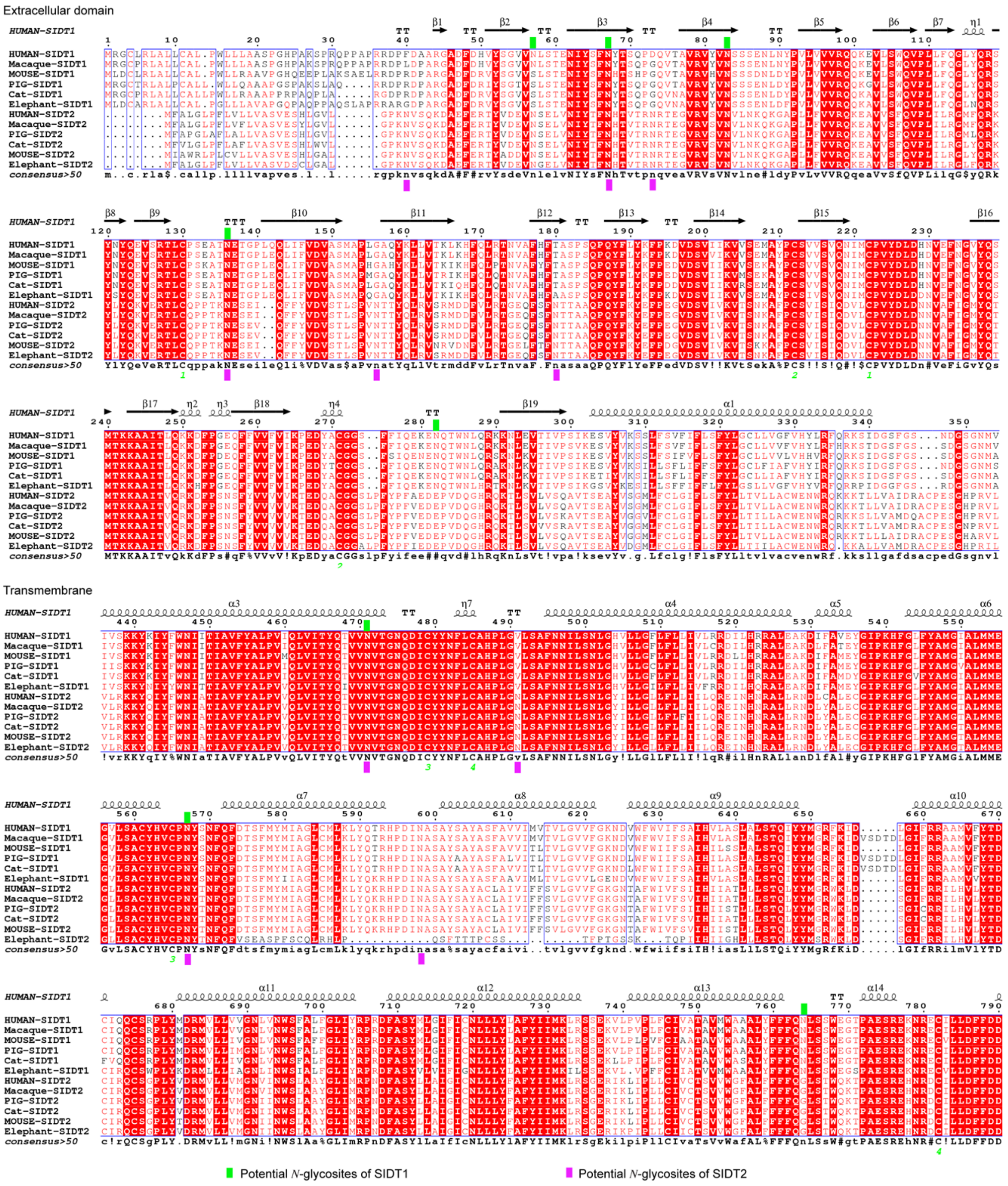

Figure S1. Potential *N*-glycosites of human SIDT1 and SIDT2. Sequence alignment of the representative sequences across different mammalian species, including human, macaque, mouse, pig, cat, and elephant. Secondary structure elements predicted by AlphaFold are indicated by

arrows for β strands and cylinders for α helices(1,2). Potential *N*-glycosites aligned to human SIDT1 and SIDT2 are highlighted in green and purple, respectively. Sequences were aligned using Clustal Omega(3) and visualized using ESPript 3.0(4).

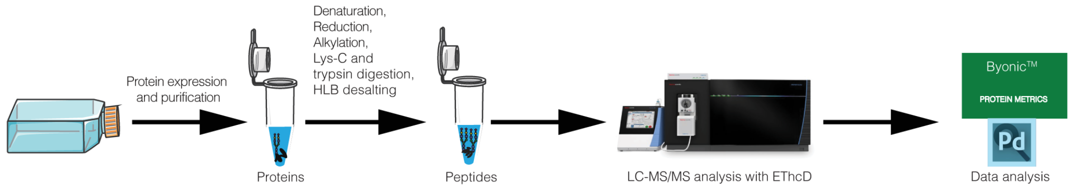

Figure S2. The workflow for site-specific *N*-glycosylation characterization of recombinant proteins. SIDT1 and SIDT2 proteins recombinantly expressed in HEK293F cells with intact *N*-glycosylation was digested into peptides and the intact glycopeptides were analyzed with LC-MS/MS using EThcD mode.

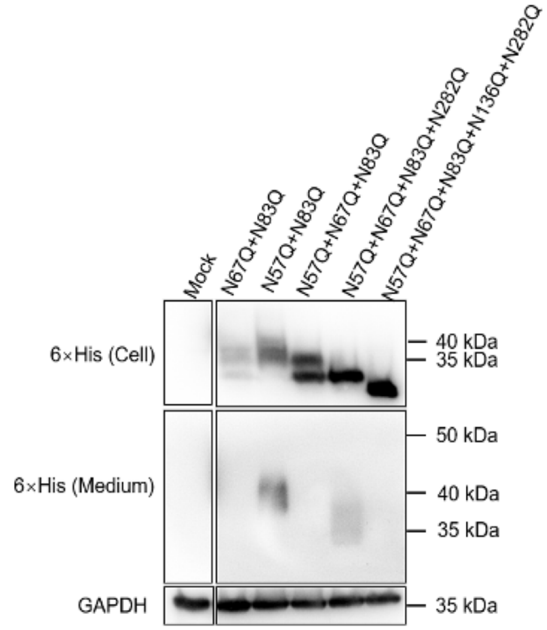

Figure S3. Effect of *N*-glycosylation on SIDT1^{ECD} protein secretion. Western blots were analyzed for lysate and medium from SIDT1ECD and combined *N*-glycosites mutants transfected HEK293T cells. Three independent experiments were performed.

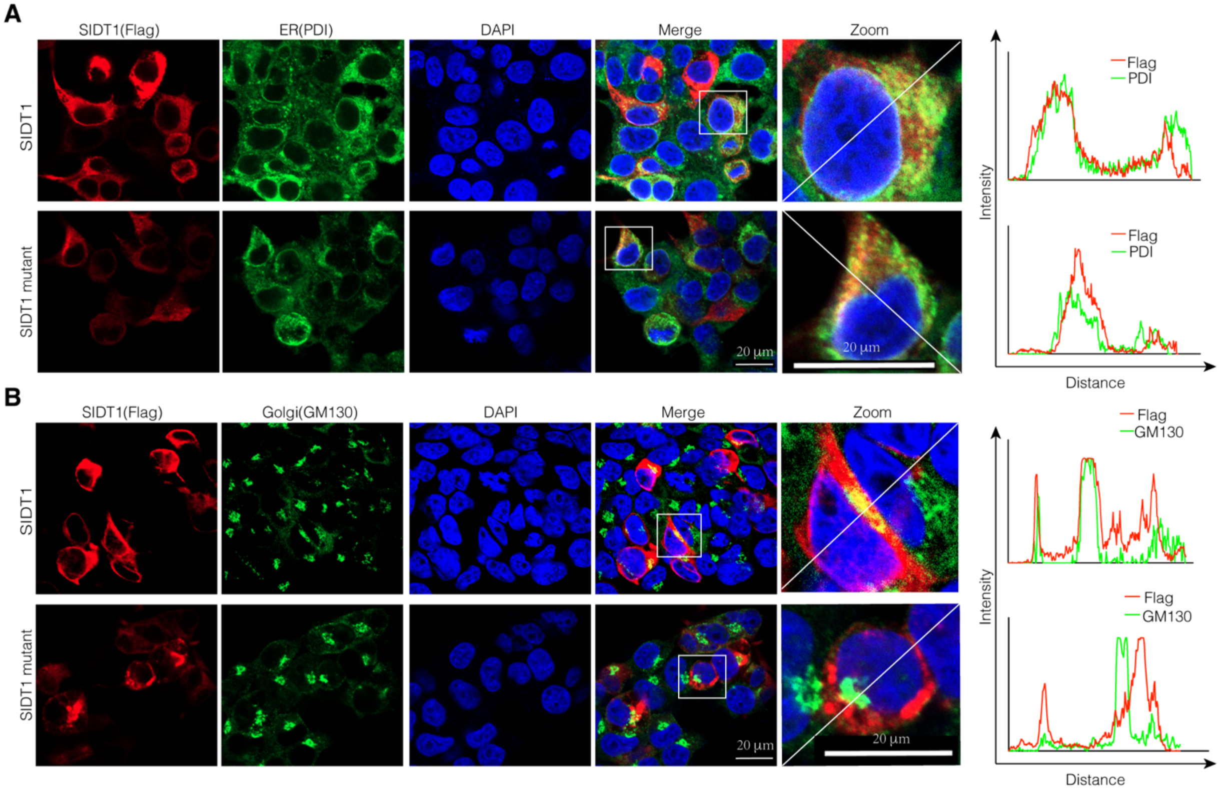

Figure S4. The effect of *N*-glycosylation on the cell surface expression of SIDT1. **A** Representative confocal microscopy images showing the ER localization in cells expressing wild-type SIDT1 and SIDT1 mutant variant. ER marker protein disulfide isomerase (PDI) (green) and anti-Flag (red) antibodies were used. Scale bar, 20 μm. **B** Representative confocal microscopy images showing the Golgi localization in cells expressing wild-type SIDT1 and SIDT1 mutant variant. Golgi marker GM130 (green) and anti-Flag (red) antibodies were used. Scale bar, 20 μm. Three independent experiments were performed.

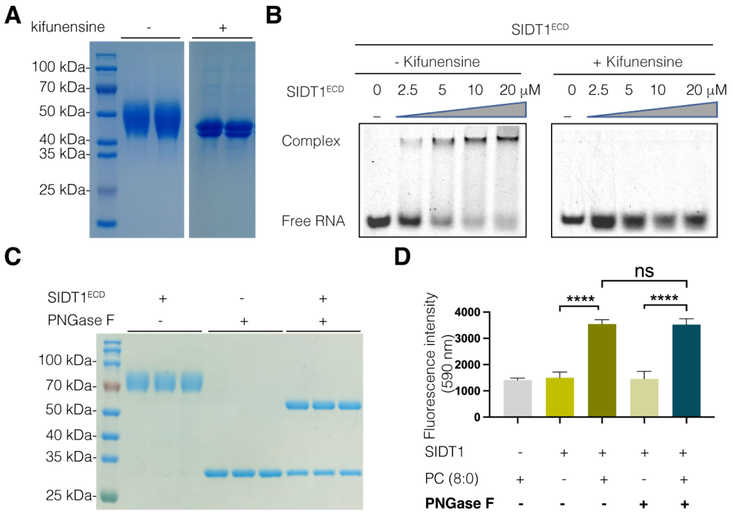

Figure S5. Effect of *N*-glycosylation on RNA binding of SIDT1. A SDS-PAGE analysis of purified kifunensine-treated and -untreated SIDT1^{ECD}. The Kifunensine-treated SIDT1^{ECD} proteins were obtained by adding kifunensine during protein expression. **B** RNA binding of SIDT1^{ECD} obtained from kifunensine-treated or -untreated HEK293F cells as determined by EMSA. The final protein concentrations of SIDT1^{ECD} in lanes 1-5 are 0, 2.5, 5, 10, and 20 μM, respectively, and the final 5'-FAM-labeled ssRNA concentration is 2.5 μM. Complex: bound; Free ssRNA: unbound. **C** SDS-PAGE analysis of PNGase F-treated and -untreated SIDT1^{ECD}. Deglycosylated SIDT1^{ECD} proteins were obtained by the deglycosylation with PNGase F. **D** Analysis of the phospholipase activity of PNGase F-treated and -untreated SIDT1. Deglycosylated SIDT1^{ECD} proteins were obtained by the deglycosylation with PNGase F. Phospholipase activity was determined by comparing the fluorescence readings of test samples to a standard curve. Prism 8 software (GraphPad) was used to perform statistical analysis and determine significant differences between samples and controls. Three independent experiments were performed.

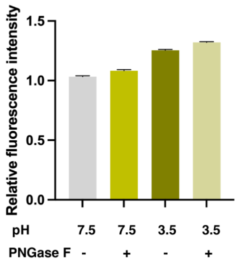

Figure S6. Effect of *N*-glycosylation on SIDT1 mediated RNA uptake. Cell viability of PNGase F-treated (+) and untreated (−) PANC1 cells was determined by CCK-8 assay. The optical density (OD) value of each well was measured at 450 nm. Three independent experiments were performed.

REFERENCES

1. Tunyasuvunakool, K., Adler, J., Wu, Z., Green, T., Zielinski, M., Zidek, A., Bridgland, A., Cowie, A., Meyer, C., Laydon, A., Velankar, S., Kleywegt, G. J., Bateman, A., Evans, R., Pritzel, A., Figurnov, M., Ronneberger, O., Bates, R., Kohl, S. A. A., Potapenko, A., Ballard, A. J., Romera-Paredes, B., Nikolov, S., Jain, R., Clancy, E., Reiman, D., Petersen, S., Senior, A. W., Kavukcuoglu, K., Birney, E., Kohli, P., Jumper, J., and Hassabis, D. (2021) Highly accurate protein structure prediction for the human proteome. *Nature* **596**, 590-596
2. Varadi, M., Anyango, S., Deshpande, M., Nair, S., Natassia, C., Yordanova, G., Yuan, D., Stroe, O., Wood, G., Laydon, A., Zidek, A., Green, T., Tunyasuvunakool, K., Petersen, S., Jumper, J., Clancy, E., Green, R., Vora, A., Lutfi, M., Figurnov, M., Cowie, A., Hobbs, N., Kohli, P., Kleywegt, G., Birney, E., Hassabis, D., and Velankar, S. (2022) AlphaFold Protein Structure Database: massively expanding the structural coverage of protein-sequence space with high-accuracy models. *Nucleic Acids Res* **50**, D439-D444
3. Corpet, F. (1988) Multiple sequence alignment with hierarchical clustering. *Nucleic Acids Res* **16**, 10881-10890
4. Robert, X., and Gouet, P. (2014) Deciphering key features in protein structures with the new ENDscript server. *Nucleic Acids Res* **42**, W320-324
